# Comparative Analysis of Thrombin Calibration Algorithms and Correction for Thrombin-α2macroglobulin Activity

**DOI:** 10.3390/jcm9103077

**Published:** 2020-09-24

**Authors:** William C. Chang, Joseph W. Jackson, Kellie R. Machlus, Alisa S. Wolberg, Mikhail V. Ovanesov

**Affiliations:** 1 Center for Biologics Evaluation and Research, U.S. Food and Drug Administration, Silver Spring, MD 20993, USA; willcchang@gmail.com (W.C.C.); Joseph.Jackson@fda.hhs.gov (J.W.J.); 2 Department of Pathology and Laboratory Medicine, University of North Carolina at Chapel Hill, Chapel Hill, NC 27514, USA; kmachlus@bwh.harvard.edu; 3 Department of Pathology and Laboratory Medicine and UNC Blood Research Center, University of North Carolina at Chapel Hill, Chapel Hill, NC 27514, USA; alisa_wolberg@med.unc.edu

**Keywords:** blood coagulation factors, blood coagulation tests, calibration, plasma, thrombin

## Abstract

Background: The thrombin generation (TG) test is useful for characterizing global hemostasis potential, but fluorescence substrate artifacts, such as thrombin-α2macroglobulin (T-α2MG) signal, inner filter effect (IFE), substrate consumption, and calibration algorithms have been suggested as sources of intra- and inter-laboratory variance, which may limit its clinical utility. Methods: Effects of internal vs. external normalization, IFE and T-α2MG on TG curves in normal plasma supplemented with coagulation factors, thrombomodulin, and tissue factor were studied using the Calibrated Automated Thrombinography (CAT; Diagnostica Stago, Parsippany, NJ, USA) and in-house software. Results: The various calibration methods demonstrated no significant difference in producing TG curves, nor increased the robustness of the TG assay. Several TG parameters, including thrombin peak height (TPH), produced from internal linear calibration did not differ significantly from uncalibrated TG parameters. Further, TPH values from internal linear and nonlinear calibration with or without T-α2MG correction correlated well with TPH from external calibration. Higher coefficients of variation (CVs) for TPH values were observed in both platelet-free and platelet-rich plasma with added thrombomodulin. Conclusions: Our work suggests minimal differences between distinct computational approaches toward calibrating and correcting fluorescence signals into TG levels, with most samples returning similar or equivalent TPH results.

## 1. Introduction

The clinical application of the thrombin generation (TG) assay has been suggested by multiple research studies, and it is often used for bionanalytical assessment in clinical trials [1,2]. The TG assay, while uniquely sensitive to the mechanism of action of numerous drugs developed for treatment of bleeding and thrombotic diseases [3,4,5,6,7], remains hard to standardize and validate for routine use outside of the expert central laboratories [8,9,10,11,12,13]. Modern automated TG assays are based on fluorogenic peptide substrates which release fluorophore in a relatively slow reaction with thrombin [11,14,15,16,17,18]. The fluorogenic substrate, rather than a color changing substrate, is used to overcome absorbance interference caused by formation of fibrin clots, allowing for the detection of thrombin activity in plasma during and after clotting. Slow reactivity to thrombin is important because it ensures slow substrate consumption through the end of TG reaction [14]. 

The convenience of fluorogenic substrates comes at the price of artifacts that are suspected to distort the assay results (see Table 1). One type of artifact affects the biochemical reactions, e.g., slow substrate added in excess will act as a competitive inhibitor of thrombin, both reducing access to physiological substrates and protecting thrombin from irreversible inhibitors [19]. The apparent TG signal can be overestimated even further due to other enzymes which cleave most synthetic thrombin substrates, such as Factor (F) Xa [20] or, in fibrinolysis experiments, tissue plasminogen activator. Such artifacts are inseparable from the substrate properties used in the assay, and, arguably, they cannot be corrected by any means except by substituting for a different kind of substrate reporting approach, e.g., faster substrates [21].

Another group of artifacts, which is limited to the interaction of fluorophore and substrate with the recording system, can be addressed through additional calibration, mathematical correction, or both [15,16,22,23,24,25,26,27,28,29] (see Table 2). For example, variability between and within fluorescence readers may require calibration of the instrument to ensure consistent conversion of the fluorescence signal into units of thrombin activity [29]. Similarly, optical properties of plasma samples, such as quenching of fluorophore, may call for preparation of calibrating fluorophore solutions in matched plasma samples [22,23,26]. Fluorogenic substrate consumption by a biologically inactive thrombin-α2macroglobulin (T-α2MG) complex can be corrected through subtraction of residual background signals by mathematical algorithms [28]. The underreporting of thrombin activity due to fluorogenic substrate depletion near the end of the TG experiment can be corrected by solving the Michaelis–Menten equation [22,23,26]. Finally, fluorescence self-quenching via a so-called inner filter effect (IFE) and resulting non-linearity of responses to high fluorophore concentrations can be corrected by calibration curves [22,23,27] or internal thrombin calibration in the Calibrated Automated Thrombinography (CAT) [16]. 

Commercial TG kits and in-house methods of research groups differ in their application of correction algorithms, and the impact of this variability on the assay performance remains unknown. Early studies of fluorogenic TG assays used a calibrated concentration of fluorophore as an assay readout [14,17] that, in conjunction with the Michaelis–Menten equation, could be used to convert the substrate consumption rate into thrombin activity [26]. A calibration curve made with purified thrombin was subsequently introduced to convert the substrate consumption rate into thrombin activity by means of a calibration coefficient [25,30,31]. However, Hemker et al. [16] demonstrated that the calibration coefficient, linking the fluorescence increase with the thrombin activity, changes over the course of the reaction due to the IFE and substrate depletion. To compensate for this, the non-linearity of the fluorescence-thrombin relationship can be estimated with a T-α2MG calibrator, tested in parallel with the TG samples, allowing for the correction of TG curves on matching (internal to the assay) calibrator signals, i.e., CAT approach [15,16]. Thus, all TG analysis algorithms include a calibration step where relative fluorescence units recorded by the instrument are converted into the concentrations of fluorophore or thrombin, which is achieved by either a fluorophore or thrombin calibrator (a sample with known substrate-cleaving activity, expressed in nM thrombin). The calibrator can be either internal and tested in parallel with the sample of patient plasma, or external to the assay, i.e., tested in a separate experimental run.

The relative benefits of various calibration and correction approaches on the TG parameters are not immediately obvious. With the exception of the area under the TG curve (AUC, also known as Endogenous Thrombin Potential or ETP), which is reduced after T-α2MG residual signal is subtracted, TG curve parameters may be affected by calibration and correction only marginally. Indeed, time parameters of the TG curve, i.e., Lag Time (LT) and Time to Thrombin Peak (TTP) should not be affected by calibration. Further, as the substrate is slow-reacting, one would expect the changes in both substrate consumption and IFE to be limited. 

In this study, we investigated raw fluorescence kinetic data on model plasma samples with a wide range of procoagulant potentials to compare internal vs. external calibration and Michaelis–Menten calculations with non-linear correction approaches with T-α2MG correction. Furthermore, we compared the same raw TG and thrombin calibrator data processed by multiple calibration and artifact correction algorithms, over a wide range of TG potentials.

## 2. Experimental Section

### 2.1. Principles of TG Data Analysis

**Figure 1:**
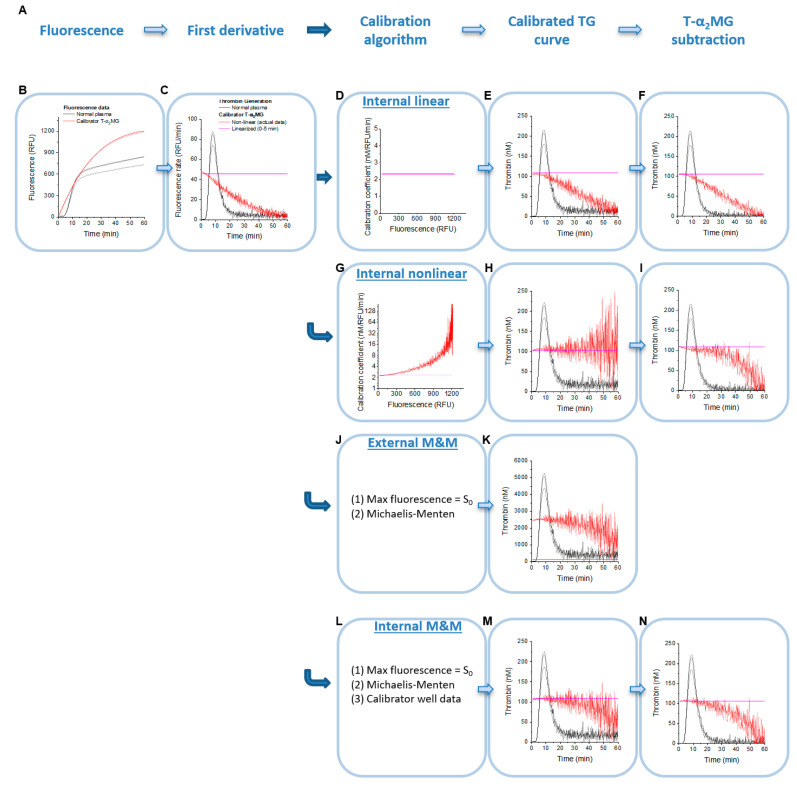
Application of calibration algorithms and thrombin-α2macroglobulin (T-α2mg) correction to fluorescence data from thrombin calibrator (red lines) and normal pooled platelet free plasma (PFP, gray lines) triggered with 1 pM TF. This experiment was performed on the CAT instrument and triggered with PPP low reagent. (**A**) The sequence of data analysis procedures as applied by the in-house software. (**B**) Raw fluorescence time-course data for pooled PFP samples and thrombin calibrator (n = 3 replicates each). (**C**) First derivative of fluorescence time course (rate calibration of substrate consumption rate). (**D**,**G**,**J**,**L**): Calibration approaches applied to data in (C): (**D**) Internal linear calibration approach for relative fluorescence units (RFU) per minute into nM of thrombin (calibration coefficient for conversion is the same regardless of the fluorescence level), (**G**) internal nonlinear calibration (takes into consideration the non-linearity of calibration coefficient as derived from the thrombin calibrator curve), (**J**) external Michaelis–Menten (M&M) formula (traditional approach based on previously established *k_cat_* and K_m_), and (**L**) internal M&M (where *v_max_* is calculated from the respective calibrator wells). (**E**,**H**,**K**,**M**): TG curves obtained by the indicated calibration of the first derivative curves in (**C**). (**F**,**I**,**N**): TG curves after T-α2mg subtraction correction. Note: external M&M with T-α2mg subtraction was not calculated because this method failed to recover expected calibrator activity.

Our study was based on a series of previously published experiments [32] from which we had both raw fluorescence curves and the results of analysis using commercially available software from Calibrated Automated Thrombinography (CAT, Diagnostica Stago, Parsippany, NJ, USA). To apply different correction and calibration algorithms, or their combinations, we used our in-house software (described below). Several parameters needed for TG data processing by the Michaelis–Menten algorithm, e.g., initial concentrations of substrate (420 µM), calibrator (0.105 µM in most experiments) and *K_m_* for the thrombin-substrate interaction, were known from the assay protocol and from the literature, respectively [14,16,26]. The remaining parameters were estimated from calibrator curves, e.g., initial slope and *k_cat_*. These parameters were then used to analyze raw data side-by-side with various correction algorithms (Figure 1A). As a reference, CAT software was used, which returns results using corrections for the IFE, substrate consumption, and T-α2MG. 

### 2.2. Thrombin Generation Experiments

TG was measured [32] by a commercially-available system [16] in a single pool of normal platelet-free plasma (PFP) and, in some experiments, in single donor platelet-rich plasma (PRP) samples (one plasma sample in all wells of a 96-well plate for each assay run). PRP samples were adjusted for the platelet count of 150,000/μL by dilution with autologous PFP. 

Plasma samples were spiked with 100% or 300% levels (i.e., to reach double or quadruple the normal level, respectively) of coagulation factors V, VIII, IX, X, XI, I (fibrinogen), and II (prothrombin) to generate a range of normal-to-highly procoagulant samples. Half of the experiments were performed in the presence of 5 nM thrombomodulin (TM), at a concentration which was sufficient to lower the procoagulant potential of normal plasma samples by ~70%. For the purposes of TG algorithm evaluation, we considered TM experiments as model of hypocoagulant (bleeding) conditions. Activation of coagulation in PFP was triggered by 1 pM tissue factor (TF) and 4 µM procoagulant lipids (PL, PPP-Reagent LOW, Stago). PRP was tested with 1 pM TF.

### 2.3. Analysis Algorithms and Software

TG parameters were calculated both using CAT’s Thrombinoscope software version 3.0.0.29 (Thrombinoscope BV, Maastricht, The Netherlands) and an in-house software package using OriginPro (OriginLab, Northampton, MA, USA; the package is available from us upon request) described previously [29]. For this study, the in-house software was upgraded with an ability to use previously-described algorithms for data analysis by the CAT approach [15,16], external calibration curve [25,31], and Michaelis–Menten approach [22,26,27] (summarized in Table 2).

### 2.4. Internal Linear Thrombin Calibration

In our dataset [32], assays were internally calibrated using wells with a T-α2MG preparation calibrated in nM of thrombin. For linear slope calibration with the thrombin calibrator, i.e., for calibration that does not take the IFE and substrate consumption into account, the slope was estimated using linear regression of the beginning of the calibrator replicate wells’ concatenated fluorescence curves (6 min after the start of the run) (Figure 1B,C). The following formula was used to calculate calibrated TG curves:

(1) $$Thrombin_{calibrated}\left(t\right)=\frac{Calibrator_{0}}{k_{linear}}\times \frac{\partial \;RFU\;\left(t\right)}{\partial \;t}$$

where *RFU (t)* is fluorescence in relative fluorescence units (*RFU*) in the sample well as a function of time; *k_linear_* is the linear fitting coefficient of the thrombin calibrator well graph *RFU* (*t*); *Calibrator*_0_ is the concentration of thrombin calibrator in [nM] of thrombin; and $\frac{\partial \;RFU\;\left(t\right)}{\partial \;t}$ is rate of substrate consumption in [RFU/min].

### 2.5. Internal Non-Linear Thrombin Calibration

For nonlinear (i.e., CAT) correction (Figure 1G–I), a calibration curve fitting approach was used in which a plot of fluorescence versus change in fluorescence was fitted to a cubic polynomial; these coefficients were used to translate fluorescence changes into units of thrombin essentially as described in an internally calibrated TG approach [16]. Specifically,

(2) $$Thrombin_{calibrated}\left(t\right)=\frac{Calibrator_{0}}{a\;\times RFU{\left(t\right)}^{3}+\;b\;\times RFU{\left(t\right)}^{2}+c\;\times RFU\left(t\right)+d}\times \frac{\partial \;RFU\;\left(t\right)}{\partial \;t}$$

where *a*, *b*, *c*, and *d* are cubic polynomial fitting parameters of the substrate consumption rate $\frac{\partial \;RFU\;\left(t\right)}{\partial \;t}$ vs. fluorescence in the thrombin calibrator well; and *Calibrator*_0_ is the concentration of thrombin calibrator in [nM] of thrombin.

### 2.6. External Linear and Non-Linear Thrombin Calibration

For external calibration, calibrator well data pooled from all analyzed assay runs (except runs with low fluorescence, see Results) were used to calculate regression curves. Linear regression slopes were averaged, while for nonlinear correction, calibrator data from all analyzed runs were fitted together as a single cubic polynomial.

### 2.7. Determination of K_m_ and k_cat_ for Hydrolysis of Z-GGR-AMC by Active Site-Titrated Thrombin

For the Michaelis–Menten equation method, *K_m_* and *k_cat_* for thrombin hydrolysis of the fluorogenic substrate Z-GGR-AMC were determined on a Tecan GENios microplate reader (Tecan, Durham, NC, USA) essentially as described previously by Nagashima [26].

### 2.8. External and Internal Michaelis–Menten Calibration

To calculate thrombin activity using the Michaelis–Menten formula, the uncalibrated fluorescence signal *RFU (t)* was converted to the calibrated signal *AMC (t)* under the assumption that the highest fluorescence value *RFU_max_* in the calibrator well is equal to the initial substrate concentration *S*_0_ (420 µM), i.e., the fluorescence of the fully consumed substrate:

(3) $$AMC\left(t\right)\;=\;\frac{RFU\;\left(t\right)\;}{RFU_{max}}\times S_{0}$$

Furthermore, the Michaelis–Menten formula with substrate consumption was used:

(4) $$Thrombin_{calibrated}\left(t\right)\;=\;\frac{S_{0}-\;AMC\;\left(t\right)\;+K_{m}\;}{(S_{0}-AMC\;\left(t\right))\;\times \;k_{cat}}\times \frac{\partial \;AMC\;\left(t\right)}{\partial \;t}$$

where *S*_0_—*AMC(t)* is the current concentration of substrate; $\frac{\partial \;AMC\;\left(t\right)}{\partial \;t}$ is the substrate consumption rate; and *K_m_* and *k_cat_* are Michaelis and catalytic constants, respectively.

Two types of Michaelis–Menten formulas were used. A traditional approach based on previously established (i.e., external) *k_cat_* value (Figure 1J). Alternatively, in the internal Michaelis–Menten approach, an apparent *k_cat_^apparent^* was calculated from the respective calibrator wells (Figure 1L) using a known concentration of thrombin calibrator from the following equation:

(5) $$Calibrator_{0}\;=\;\frac{S_{0}+K_{m}\;}{S_{0}\;\times \;k_{cat^{apparent}}}\times {\left(\frac{\partial \;AMC}{\partial \;t}\right)}_{max}$$

where ${\left(\frac{\partial \;AMC}{\partial \;t}\right)}_{max}$ is the highest rate of substrate consumption in the calibrator well.

## 3. Results

### 3.1. Effect of Internal Linear and Non-Linear Thrombin Calibration and Michaelis–Menten Formula on Calibrator and Normal TG

All thrombin calibration approaches start with the first derivative of fluorescence vs. time-course data (Figure 1A–C). As expected, signals in the calibrator well sharply declined almost immediately after the start of the experiment, demonstrating a strong non-linear effect of substrate depletion and IFE. Three thrombin calibration methods were studied in this work: linear calibration (Figure 1D), non-linear (CAT-like, Figure 1G) and both external and internal Michaelis–Menten formula-based corrections for substrate depletion (Figure 1K,M, respectfully). In some cases, background T-α2MG values were also subtracted (Figure 1F,I,N).

Applying linear thrombin calibration converted relative fluorescence units per minute (RFU/min) into thrombin concentration units without changing the shape of TG curve (Figure 1D–F). Applying non-linear calibration corrected for the decline in, and effectively linearizes, the thrombin calibrator well signal (Figure 1G–I). 

**Figure 2:**
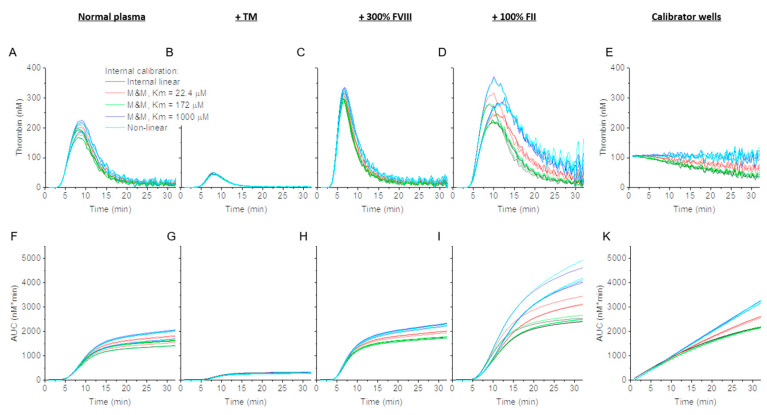
TG after internal linear, internal non-linear, and internal Michaelis–Menten (M&M) calibration in normal plasma supplemented with coagulation factor (F) II, FVIII, or thrombomodulin (TM). Representative CAT experiment in PFP triggered with 1 pM TF calibrated with the indicated method: internal linear (black), internal non-linear (cyan), or internal M&M (red: Km = 22.4 µM [26], green: Km = 172 µM [14], and blue: Km = 1000 µM). (**A**–**E**) TG curves in normal plasma supplemented with TM, 300% FVIII and 100% FII. (**F**–**K**) Area under the curve (AUC) as a function of added coagulation factor level (**E**,**K**). Curves from calibrator wells.

While the nonlinear correction method employs empirical data from internal thrombin calibrator wells to address nonlinearity that may be caused by either IFE or substrate consumption, substrate consumption itself can be corrected by using the constants of interaction between thrombin and substrate, i.e., the Michaelis–Menten formula. Assuming no IFE contribution, the maximal plateau of fluorescence signals at the end of the experiment corresponds to fully consumed substrate concentration. An attempt to determine thrombin activity in a calibrator well (a known constant thrombin activity of 105 nM) using an experimentally determined *K_m_* and *k_cat_* pair (1000 µM/0.35 s^−1^, determined in a separate experiment, i.e., “external” Michaelis–Menten) resulted in ~23 times higher thrombin calibrator values (Figure 1J,K). We also used *K_m_*/*k_cat_* constants reported in [26] (172 µM/1.2 s^−1^) and [14] (22.4 µM/0.53 s^−1^), which also returned elevated calibrator values (2.6–15 fold). The inability to calculate calibrator activity indicated that either the maximal fluorescence value was biased by IFE or that the *K_m_* and *k_cat_* values were inaccurate. To bypass both problems, for each *K_m_*, we estimated “apparent-internal” *k_cat_* values of 199 min^−1^, 150 min^−1^, and 491 min^−1^ respectively, such that the thrombin calibrator signal was equal to its expected thrombin activity (105 nM) (Figure 1L,M and Figure 2A). After Michaelis–Menten application, substrate consumption correction improved the decreased calibrator signal values (Figure 1K,M) although it did not achieve complete correction as was seen in the non-linear algorithm (Figure 1H).

### 3.2. Effect of Thrombin Calibration and Correction on TG in Factor Supplemented Plasma 

TG in the presence of TM or added coagulation factors appeared, respectively, hypo- or hyper-coagulant regardless of the calibration approaches applied. For all conditions except prothrombin (FII)-supplemented plasma, TG curves produced by the linear and non-linear calibration algorithms, and Michaelis–Menten calculations were indistinguishable from one another (Figure 2A–D). For prothrombin samples, both the non-linear and M&M calibrations provided an upward adjustment of TG curves (Figure 2D). Further, Michaelis–Menten and non-linear algorithms produced similar calibration curves (Figure 2E,K). The calibration methods produced similar area under the curve (AUC) values, with the exception of the samples supplemented with prothrombin, which were adjusted upward in the non-linear and Michaelis–Menten methods (Figure 2F–I). It should be noted that the differences produced between the different calibration algorithms, especially in the prothrombin samples, are observed in the portion of the TG curve produced after the thrombin peak height (TPH) is reached, and TPH was minimally affected. The magnitude of the substrate consumption correction using “apparent-internal” *k_cat_* values depended on *K_m_*, giving higher adjustment for higher values of *K_m_*. For a wide range of TG potentials, namely TPH, obtained after coagulation factor and TM supplementation, the Michaelis–Menten algorithm, with a *K_m_* value of 1000 µM, did not differ significantly from those returned by the non-linear algorithm (Supplementary Materials, Figure S2A). Therefore, the impact of the Michaelis–Menten algorithm was not studied further. Furthermore, TPH values, regardless of calibration method, did not differ significantly from uncorrected TPH, suggesting that calibration is unnecessary (Supplementary Materials, Figure S2B).

### 3.3. Effect of Internal Calibration on TG Parameter Values in PFP 

**Figure 3:**
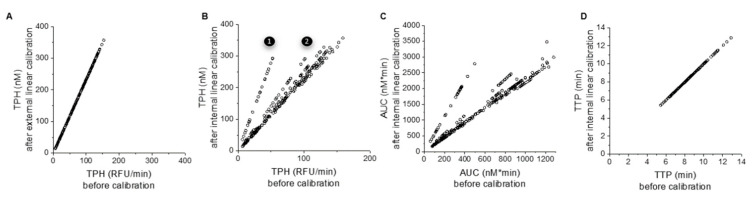
Effect of internal calibration algorithms on TG parameter values in PFP supplemented with coagulation factors and/or TM. Each dot represents a single experimental condition (e.g., NPP + 100% FVIII + TM, averaged over 3 replicates). Experiments with added FII were excluded due to unusual behavior. The graphs show correlation between uncalibrated TG parameter values (*x*-axis) and respective values after correction-calibration. (**A**) TPH before and after external linear calibration (TG curves multiplied by a calibration coefficient determined from all experimental runs). (**B**) TPH before and after internal linear calibration (where the calibration coefficient was calculated from internal calibrator wells in each experimental run). Comment: 1 and 2 denote experiments with low overall fluorescence, corrected by internal calibrator (calibrators shown in Supplementary Materials, Figure S3). (**C**) AUC before and after internal linear calibration. (**D**) TTP before and after internal linear calibration.

To compare the individual impact of correction algorithms, we used a series of 1 pM TF-activated TG experiments in which coagulation factors and TM were added to a single pool of PFP (8 runs for most conditions). Prothrombin-supplemented samples were excluded from analysis because the non-linear algorithm struggled with correcting TG curves under this condition. External linear calibration (i.e., no correction) correlated with uncalibrated TPH results (Figure 3A). In contrast, when internal linear calibration was applied, the TG parameters TPH, AUC, time to thrombin peak (TTP) (Figure 3B–D), and lag time showed good linear correlation with uncalibrated results, suggesting that this method is unnecessary. However, internal calibration revealed run-to-run variability in the calibrated signal (Supplementary Materials, Figure S1). Two assay runs (1 and 2 on Figure 3B,C) had lower fluorescence signals resulting in lower uncalibrated TG curves (Figure 3 and Supplementary Materials, Figure S1), which were subsequently corrected via internal calibration. 

### 3.4. Individual Contributions of Internal Linear/Non-Linear, and T-α2MG Corrections vs. CAT

#### 3.4.1. Effect of Internal vs. External Linear Thrombin Calibration

**Figure 4:**
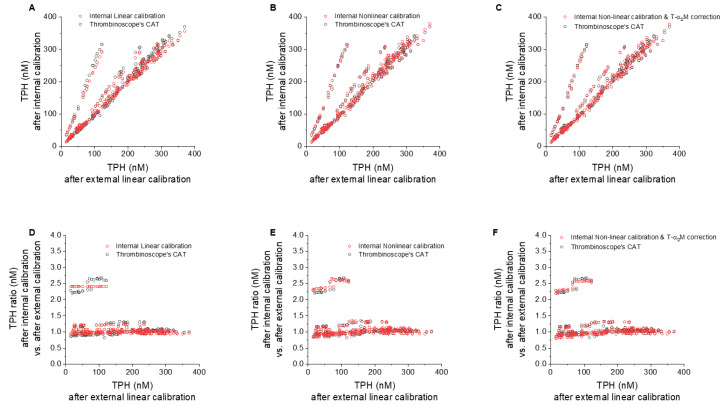
Effect of internal vs. external calibration algorithms on TPH values in PFP supplemented with coagulation factors and/or TM. Each dot represents a single experimental condition, as described in the legend for Figure 3. Red dots: analysis with our in-house software, black dots: analysis with commercial software (CAT-Thrombinoscope). (**A**–**C**) TPH after external linear calibration vs. (**A**) internal linear calibration, (**B**) internal non-linear calibration, and (**C**) internal non-linear calibration followed by T-α2MG correction. (**D**–**F**) Normalized TPH (to external linear calibration) after (**D**) internal linear calibration, (**E**) internal non-linear calibration, and (**F**) internal non-linear calibration followed by T-α2MG correction.

To compare the contribution of internal vs. external linear calibration approaches, we pooled data from multiple calibrator wells to generate one linear calibrator curve. TPHs were consistent between internal and external linear calibration (Figure 4A), deviating by no more than 8% (Figure 4D), with the exception of the two runs discussed above, which were corrected by 1.2- and 2.4-fold (Figure 4D).

#### 3.4.2. Effect of Nonlinear Correction

Only a small difference was observed between the internal linear calibration and commercial CAT results (Figure 4A,D). Non-linear calibration without T-α2MG subtraction almost overlapped with CAT TPH results (Figure 4B,E), again with the exception of the two runs outlined previously.

#### 3.4.3. Effect of T-α2MG Correction 

Subtracting background T-α2MG values had a small effect on normal TG curves (Figure 1F,I,N). Similarly, a negligible effect was seen on TPH values in all samples in the presence of TM or added coagulation factors as evidenced from comparison of CAT results with the non-linearly corrected TPH values with (Figure 4B,E) or without (Figure 4C,F) T-α2MG subtraction. 

### 3.5. Effect of Corrections on Run-to-Run and within-Run Variability

**Figure 5:**
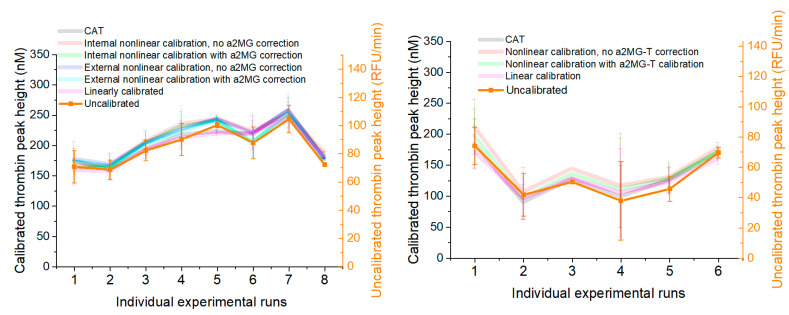
Impact of external and internal correction algorithms on run-to-run consistency in TPHs in normal PFP and PRP. **Left panel**: Normal PFP triggered with 1 pM TF; Right panel: Normal PRP triggered with 1 pM TF. Left axis: Calibrated TPH values for the normal plasma condition of each run are shown. The uncalibrated TPH axis (**right**) is scaled using the linear regression slope obtained from the composite external calibration curve.

**Figure 6:**
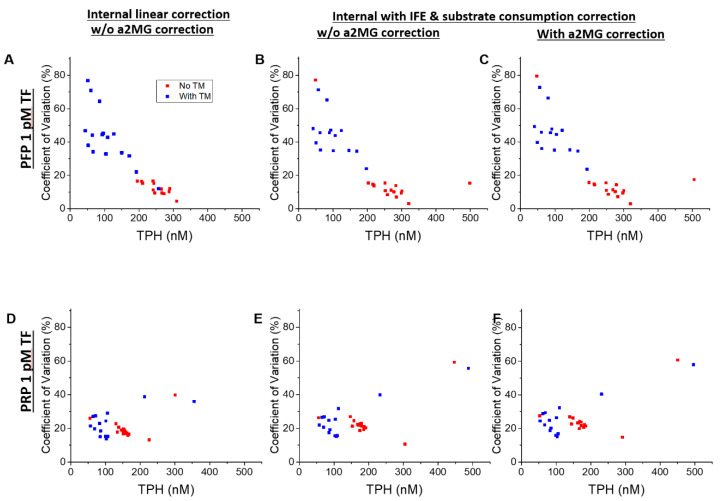
Correlation between run-to-run variation and TPH. Coefficients of variation (CVs) between runs of the same condition were calculated and plotted against the average TPH for their respective conditions. Each dot represents a single experimental condition (normal plasma plus coagulation factor). Conditions with added TM are plotted in blue; conditions without added TM are plotted in red. (**A**–**C**) PFP 1 pM TF; (**D**–**F**) PRP 1 pM TF. (**A**,**D**) internal linear correction; (**B**,**E**) internal nonlinear correction (i.e., IFE and substrate consumption corrections); (**C**,**F**) internal nonlinear and T-α2MG corrections.

The effect of internal and external linear and nonlinear corrections with or without T-α2MG subtraction on the run-to-run variability was studied across several assay runs. Under normal conditions, i.e., PFP and PRP without added coagulation factors or TM (Figure 5), TPH values obtained from repeated measurements gave comparable relative coefficients of variation (CVs) without correction (17.4%), internal linear correction (16.5%) and fully corrected approach (15.7%) in PFP and, respectively, 22.8%, 27.0%, and 27.0% in PRP (see Supplementary Materials, Tables S1 and S2). Low procoagulant PFP samples (i.e., samples with added TM) were much more susceptible to run-to-run variability than others and there appeared to be a negative correlation between TPH and CV (Figure 6, top row). This could be explained by an additional pipetting step (addition of TM). Alternately, there may be a biochemical threshold somewhere around this procoagulant level. These trends were not clear for PRP (Figure 6, bottom row) possibly because PRP’s run-to-run variability is mediated by the procoagulant activity of platelets, collected from a new donor for each run. CVs were similar between linear and nonlinear calibration for most conditions (Figure 6).

Within-run variability in normal plasma was found comparable by studying CVs obtained by averaging multiple (n = 2–3) plasma wells on one microplate.

TPH values calculated based solely on the fluorescence readings vary significantly between runs (with a CV of 28%, see Supplementary Materials, Table S2). This variation largely remains when calibration is applied, regardless of whether the CAT or our in-house algorithms were used. Similar ranges, though not necessarily patterns, of variation are seen across other conditions analyzed. We also assessed the outcome from pooling calibrator readings from multiple runs (Figure 5). If pipetting and dispensing are consistent from run to run, this gives the software more calibrator readings to average, thus removing one source of error; however, this method eliminates the ability for calibration to normalize against run-to-run inconsistencies. In the runs studied here, the latter concern prevails—the variation in peak heights between runs resembles that of the uncalibrated peaks, including the relatively low signal in runs 1 and 2 (with lower overall fluorescence) noted above.

## 4. Discussion

One purpose of thrombin calibration is to establish a mathematical relationship between fluorescence changes and thrombin levels. In an idealized environment, this would simply be the slope of the substrate consumption curve divided by the thrombin calibrator’s activity. However, substrate depletion and IFE cause this curve to deviate from being linear, T-α2MG can lead to the overestimation of free thrombin activity, and fluorescence differences from run to run or plasma sample to sample may result in under- or over-estimation of the thrombin signal. Mathematical treatments are being used to correct these artifacts. Although artifact correction is desirable, little evidence exists to support its benefit for measured TG assay values and their interpretation. By reanalyzing TG data generated on the commercial internally calibrated platform, we assessed the impact of several approaches used to correct fluorescence artifacts. Overall, we found that these correction algorithms have minimal impact on TG curves. 

Our study is limited because we used data from coagulation factors added to normal plasma to simulate a broad range of coagulabilities. Moreover, the effects of anticoagulants and inhibitors other than TM were not examined. Consequently, the clinical consequences of their different behaviors under TG testing are not known and would be of interest for future studies. Despite this, our work suggests the IFE, substrate consumption and T-α2MG correction algorithms are not necessary because there is little need for correction. We observed minimal differences between different computational approaches toward calibrating and correcting fluorescence signals into TG levels. Whether calibration of fluorescence substrate consumption to thrombin activity is calculated using a simple linear relationship or more complex calculations correcting for known artifacts, most samples give similar or equivalent results for TPH and AUC. Notably, supplementation of normal plasma with coagulation factors and TM obtained a wide range of TPHs in PFP samples stimulated with 1pM TF (12 to 337 nM in samples without prothrombin samples and 12 to 587 nM if samples with prothrombin). These TPH values overlapped with the range observed in: 217 healthy donors by Kremers et al. [33] (1 pM TF triggered PFP, TPH range: 41–371 nM), in severe-to-mild hemophilia donors described by van Veen et al. [34] (TPH 9–107 nM), and in patients with at least one thrombotic event and a confirmed diagnosis of inherited thrombophilia from Luna-Záizar et al. [35] (5 pM TF triggered PFP, TPH range: 21.8–499.3 nM). Although we did not analyze experiments with 5 pM TF and thus the effect of higher TF concentration is uncertain, it would be of interest for future studies.

Preliminary experiments were performed to assess the effect of antithrombin (AT)-mediated thrombin inhibition using affinity depleted AT-deficient PFP supplemented with increasing concentrations of AT concentrate (Supplemental Figure S4). The experiments were analyzed using our version of CAT algorithm allowing for correction for the IFE and substrate consumption, demonstrating a substantially elevated (2-fold) concentration of AT inhibits TG nearly fully. Reduced concentrations of AT are associated with increased TPHs and elevated TG tails distorted by experimental noise. Similarly, noisy TG curves and substrate consumption in AT deficiency were presented by Giesen et al. for hemophilia patients treated with fitusiran, a GalNAc-conjugated siRNA designed to target AT expression [36]. Future experiments of interest would be to perform studies in normal PFP samples with AT below 2 U/mL.

The insignificance of correction algorithms for the majority of tested samples in our work is likely explained by the minor artifact contribution in the original dataset, e.g., substrate is far from being consumed at the point when TG curves reached peak heights (Figure 1). Likewise, T-α2MG activity is far smaller than free thrombin activity at thrombin peak time, whereas it is equal or more after TG terminates, ultimately explaining why T-α2MG correction has a minimal effect on TPH while it contributes more to AUC/ETP. The limited difference in effects of nonlinear corrections on TPH and AUC/ETP calculations has been described in previous studies [37,38]. It should be noted that the disadvantage of linear calibration is that the T-α2MG end level apparently declines so that it will not be subtracted in an exact manner, i.e., the transition from Figure 1E to Figure 1F or from Figure 1H to Figure 1I may give a slight difference in results, which is negligible at low T-α2MG but not at higher ones.

The existence of fluorogenic artifacts is well known, yet surprisingly little work has been done to understand and revise these artifacts after the initial adoption of fluorogenic TG assays in early 2000s. First reported uses of fluorogenic TG assays were informative despite reporting fluorescence results only [17] or TG curve calculation without calibration in thrombin activity units [14]. Váradi et al. [30] ran the fluorogenic TG assay without calibration, reporting in terms of fluorescence units and minutes parameters commonly used to characterize TG curves: peak thrombin, thrombin potential, lag phase (time), and peak time. The residual effect of T-α2MG activity was observed and acknowledged [14], but not corrected, possibly because the signal was much lower than the one observed in the previously introduced chromogenic substrate-based assay [28]. 

One approach for addressing the nonlinear relationship between substrate consumption rate and thrombin activity is to use previously-determined kinetic constants for enzyme-substrate interaction. Several groups have characterized the behavior of thrombin against Z-GGR-AMC [14,16,26] and used this knowledge to correct for substrate consumption [22,26]. Nagashima [26] and Tarandovskiy et al. [22,23] calibrated against known concentrations of AMC, the fluorescent product of the substrate cleaved by thrombin, in plasma allowing for IFE correction, and used the Michaelis–Menten formula and previously calculated kinetic constants for thrombin to correct for substrate consumption. Further, they subtracted T-α2MG activity in the same manner as Hemker and Beguin [28]; however, they did not evaluate their approach against other ways of processing fluorometric TG data. Turecek et al. [25] converted fluorescence increase to thrombin concentration by using a reference curve of purified thrombin; nonlinear artifacts were not corrected for, but it was noted that T-α2MG activity would need to be corrected for the AUC/ETP values, while other parameters such as peak thrombin could be evaluated as is. 

Following their introduction in early 2000’s, different thrombin calibration and artifact correction algorithms were not compared in a side-by-side manner. De Smedt et al. [37] argued for the necessity of nonlinear calibration and correction; they compared the internally calibrated and externally calibrated TG assay approaches but did not separate the effects of correction algorithms from other experimental differences (plasma dilution, calibrator in buffer or in plasma, etc.). Similarly, Hemker et al. [39] mathematically modeled the nonlinear relationship between fluorescence increase and thrombin activity and found that corrections for these effects are minimal in normal non-defibrinated plasma. Chandler and Roshal [38] compared two software packages on a panel of normal donors and found that simple external calibration is sufficient. Gribkova et al. [40] gave further details about the approach introduced by Tarandovskiy et al. [22,23] and compared their approach by running patient samples with and without direct thrombin inhibitors in parallel with CAT. However, all previous studies only compared the results of the assays *in toto* rather than the individual effects of substrate consumption, IFE, and T-α2MG activity correction algorithms.

It must be noted that Michaelis–Menten kinetics assumes that substrate concentration is much larger than the concentration of products or enzymes. If the very phenomenon we are correcting for—substrate consumption—occurs at a significant level, this Michaelis–Menten assumption is violated. In addition, Michaelis–Menten does not account for IFE. Using previously-determined Michaelis–Menten values in our experiments returned thrombin values that were inconsistent with the known calibrator activity. To correct for this, we determined *k_cat_* empirically. This step resulted in TG curves and AUC/ETP values that were nearly identical to those calculated using empirical internal nonlinear calibration (Figure 2A–K and Supplementary Materials, Figure S2). While the Michaelis–Menten approach provides results similar to those returned by other algorithms, the assumptions made in this model are questionable. One possibility is to combine Michaelis–Menten formulae with mathematical correction of the IFE as proposed by Palmier and Van Doren [27]. Further experiments may help in the development of a TG model with a more solid theoretical basis.

Inter- and intra-laboratory variation is another known problem for TG assays [9,41,42]. The inclusion of internal calibration has been suggested to improve run-to-run variability because the fluorescence signal in the plasma well is calibrated via a calibrator well signal that is simultaneously recorded [15,16]. By pooling calibrator data from multiple runs, we studied whether the addition of internal calibrator data, which was run in plasma separate from the experimental wells introduces error or increases robustness. We found that internal and external calibration approaches produce similar results for both PFP and PRP, in both the TG parameters and in the corresponding CVs. This finding may imply that biological well-to-well and sample-to-sample variability far outweighs any variability canceled or introduced by pooling calibrator data from multiple runs. Exceptions were the experiments with relatively low fluorogenic signal, which were accurately corrected by the internal calibration algorithm (both linear and non-linear methods). Nonetheless, to validate the external non-linear calibration-based TG assay for clinical use, it should be tested with samples from hypo- and hyper-coagulant patients to establish relationships between clinical outcomes and TG parameters. New patterns or even new parameters may emerge as a greater variety of samples are tested. Indeed, further confirmation by multi-laboratory studies, similar to the studies by Perrin et al. [42] and van Paridon et al. [43] is needed to establish the best approaches to analyze and interpret TG tests in order to develop this technique for clinical use.

We should stress that the necessity of internal calibration when using one and the same plasma under different circumstances, as studied in this work, can be different from the comparison of different plasmas, such as a patients’ sample to a reference plasma. A reference well providing a calibration curve at constant T-α2MG activity may be advantageous for comparing plasmas with different optical properties due to color or fluorescence quenching. Not using internal calibration on such optically abnormal patient samples would make it difficult for the results from these patient plasmas to be expressed as a % of a normal reference plasma. Further investigation of the impact of plasma optical properties on the assay performance is therefore needed.

## 5. Conclusions

The TG assay has great potential for assessing hemostasis in both basic and applied clinical research. In addition to the potential fluorescence artifacts due to substrate consumption and the IFE, it was suggested that the calibration algorithm applied to the assay may also be a source of analytical variability. The work presented here suggests that the multiple calibration methods studied, including simple linear processes or more complex calculations, give similar or equivalent TPH results. This ultimately suggests that there are minimal differences between the distinct calibration methods.

## Figures and Tables

**Table 1:** Known artifacts caused by fluorescent substrates in TG assays.

Artifact	Description	Potential Impact (Artifacts) of TG Curve
Fluorescent signal variation between instruments	Instrument-specific characteristics of fluorescent lamp and detector	Thrombin values cannot be compared between instruments
Fluorescent signal variation between instruments between runs	Fluorescent lamp aging may cause gradual change as instrument gets older	May increase run-to-run variability in TG values determined on the same sample of plasma on different days
Variable quenching of fluorescence by plasma	Occasionally, plasma composition, e.g., hemolysis, may quench fluorescence	May result in the under-estimation of TPH, velocity and AUC
Substrate depletion in highly procoagulant samples	Fluorogenic substrate ZGGR-AMC is gradually consumed during thrombin generation	May result in the under-estimation of AUC and, in extreme cases, TPH and TTP
Nonlinearity of high fluorescence signal detection (IFE)	High concentrations of AMC are underestimated because fluorescence is quenched in a thick layer of plasma	May result in the under-estimation of AUC and, in extreme cases, TPH and TTP
Background signal due to T-α2MG activity	Biologically inactive T-α2MG complex can cleave small peptide substrates almost as efficiently as biologically active thrombin	May result in the over-estimation of AUC and, in extreme cases, TPH
Competitive inhibition of thrombin by slow fluorogenic substrate	Supra-physiological concentration of fluorogenic substrate slows thrombin inhibition and prevents access of physiological substrates (e.g., fibrinogen)	Over-estimation of TG curve and potential delay in TG initiation.
Poor substrate specificity (e.g., cleaved by FXa and tPA)	Peptide substrate can be cleaved by a variety of coagulation enzymes	Overestimation of TG curve and distortion of TG curve shape

Abbreviations: TPH, thrombin peak height; AUC, area under the TG curve; TTP, time to thrombin peak.

**Table 2:** Internal and external algorithms used for fluorescence calibration and artifact correction of TG data. Note that a mathematical algorithm of subtracting background T-α2MG activity from the calibrated and corrected TG curve is not shown here [28].

		Artifact					Evaluated in This Work	References
		Fluorescent Signal Variation between Instruments	Fluorescent Signal Variation between Runs	Variable Quenching of Fluorescence by Plasma	Substrate Consumption in Highly Procoagulant Samples	Nonlinearity of High Fluorescence Signal Detection (IFE)		
Fluorescence calibration external to sample plasma	Linear (slope ratio) calibration	Corrected by thrombin calibration coefficient ^1^	Not corrected		Not corrected		Yes ^2^	Turececk et al. [25]
	Nonlinear calibration (slope ratio calibration, corrected for level of fluorescence ^3^)				Slope ratio calibration, corrected for level of fluorescence		Yes	Not tried before
	M&M formula ^4^	Corrected by fluorescence calibration coefficient ^1^			Corrected by M&M formula	Corrected by IFE formula ^6^ or calibration curve ^7^	No	Nagashima [26]
	M&M formula + IFE correction							Palmier & Van Doren [27]
Fluorescence calibration internal to sample plasma	Linear calibration	Corrected by thrombin calibration coefficient ^4^			Not corrected		Yes	Not tried before
	Nonlinear calibration ^3^				Corrected using thrombin calibrator’s TG curve		Yes	Hemker et al. [15,16]
	M&M formula	Corrected by fluorescence calibration coefficient ^1^			Corrected by M&M formula	Not corrected	Yes	Nagashima [26]
	M&M formula + IFE curve ^5^					Corrected by IFE calibration curve ^6^	No	Tarandovskiy et al. [22,23]
	M&M formula + IFE formula ^5^					Corrected by IFE formula ^5^		Palmier & Van Doren [27]

^1^ Thrombin calibration coefficient is calculated as ratio of relative rate of substrate consumption (RFU/min) to concentration of thrombin calibrator (nM thrombin); ^2^ One-point calibration only; ^3^ Part of CAT algorithm; ^4^ Part of CAT algorithm; ^5^ Fluorescence calibration coefficient is calculated as the ratio of measured relative fluorescence signal (RFU) to concentration of fluorophore calibrator (µM AMC). Coefficient can be derived from one or several calibration points. ^6^ IFE formula was described in the literature in application to fluorogenic substrates. Fluorescence coefficient is corrected for level of fluorescence; ^7^ IFE calibration curve approach is based on a nonlinear calibration curve in which fluorescence coefficient is different for each level of fluorescence.

## Supplementary Materials

The following are available online at https://www.mdpi.com/2077-0383/9/10/3077/s1, Table S1: Effect of correction algorithms on coefficient of variation for normal plasma samples, Table S2: Coefficients of variation for TPHs for all conditions analyzed, Figure S1: Uncalibrated and calibrated thrombin generation curves in normal pooled plasma with or without 200% prothrombin, Figure S2: Effect of calibration on TPH in NP supplemented with coagulation factors, Figure S3: Relative adjustment in % of TPH after internal calibration vs. external calibration, and Figure S4: Effect of antithrombin (AT) addition to AT-deficient plasma on TG.
