# Supplementary material for: Comparative Analysis of Thrombin Calibration Algorithms and Correction for Thrombin-α2macroglobulin Activity
Source: J Clin Med. 2020 Sep 24;9(10):3077. doi: 10.3390/jcm9103077 (PMC7650706; doi:10.3390/jcm9103077)
Supplement: Supplementary file 1 [file jcm-09-03077-s001.zip › Supplemental Figure S1.docx]

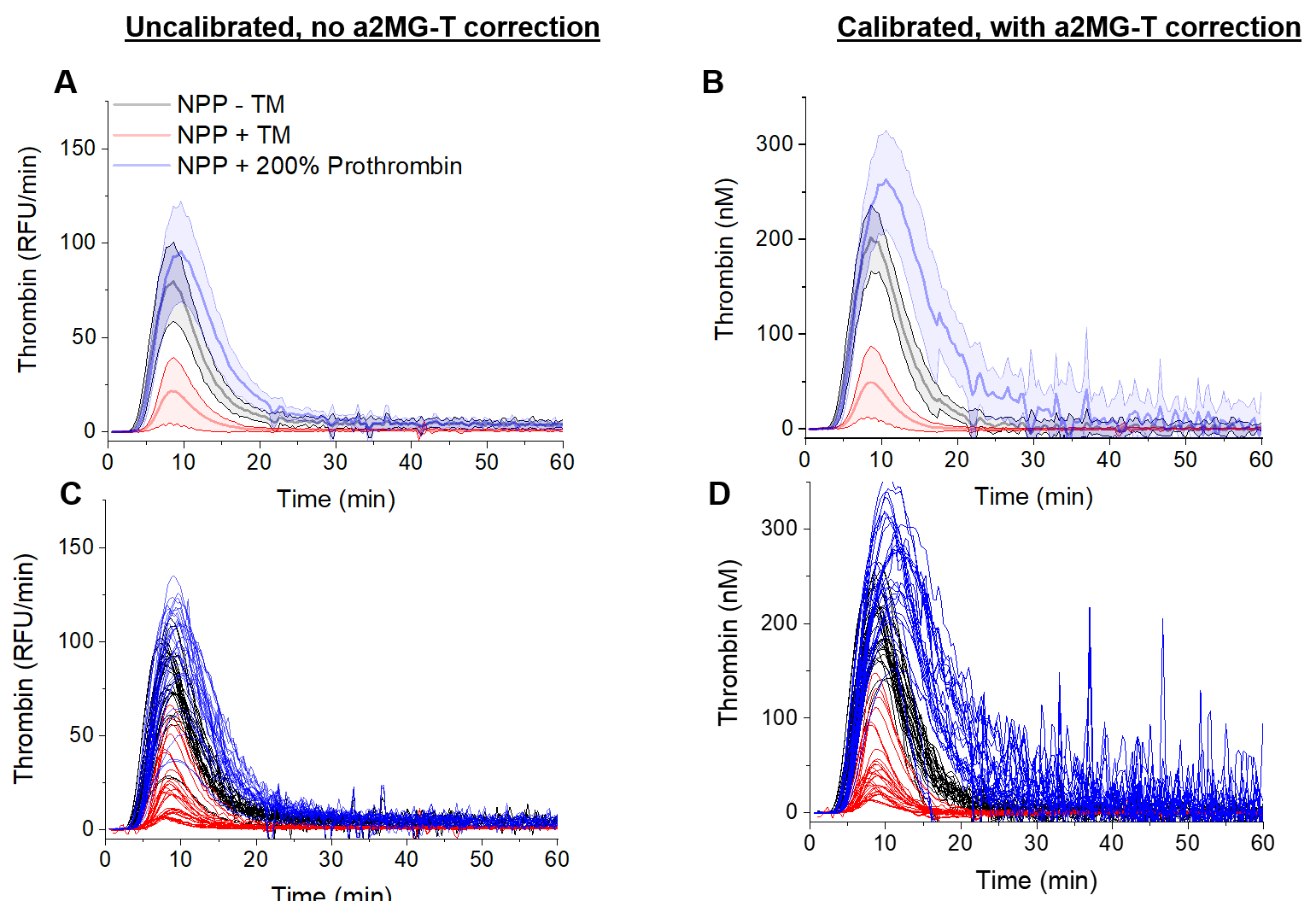


**Supplemental Figure S1. Uncalibrated and calibrated thrombin generation curves in normal pooled plasma with or without 200% prothrombin.** TG curves in normal pooled plasma treated with TM (red) or without (black), or supplemented with 200% prothrombin (blue). **Panel A and C.** Uncalibrated TG curves. **Panel B and D.** Calibrated TG curves with with T-α2MG correction.
