# Supplementary material for: Comparative Analysis of Thrombin Calibration Algorithms and Correction for Thrombin-α2macroglobulin Activity
Source: J Clin Med. 2020 Sep 24;9(10):3077. doi: 10.3390/jcm9103077 (PMC7650706; doi:10.3390/jcm9103077)
Supplement: Supplementary file 1 [file jcm-09-03077-s001.zip › Supplemental Figure S2.docx]

**
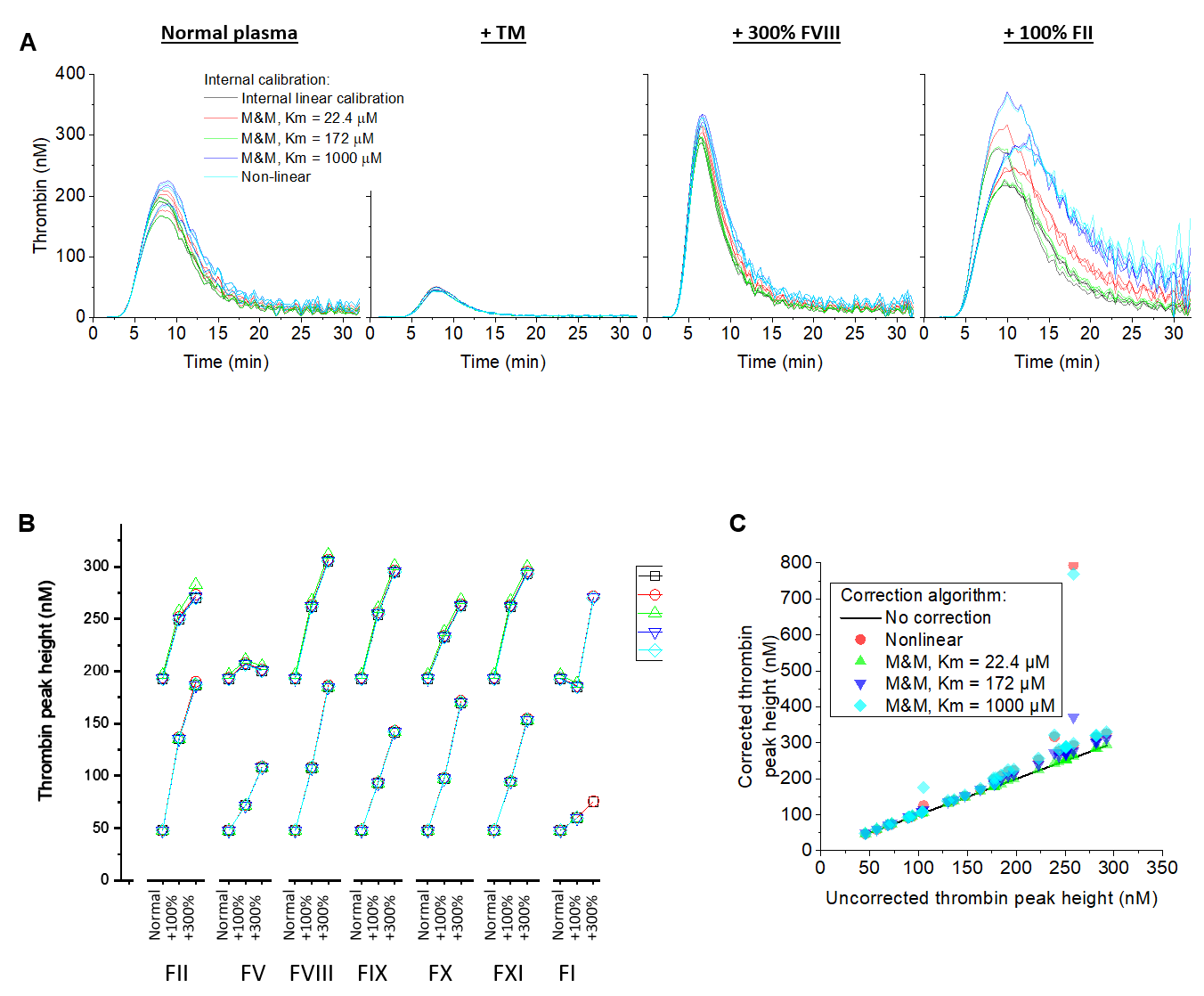
**

**Supplemental Figure S2. Effect of calibration on TPH in NP supplemented with coagulation factors.** Representative TG experiment triggered by 1 pM TF with the indicated calibration method applied. **Left.** Thrombin peak height (TPH) as a function of added coagulation factor level. Top row curves: experiments without TM; bottom row curves: experiments with TM. **Right.** Correlation between externally calibrated (uncorrected) TPH values and internally corrected TPH values. Each dot represents a single experimental condition (e.g., NPP + 200% factor VIII + thrombomodulin).
