# Supplementary material for: Comparative Analysis of Thrombin Calibration Algorithms and Correction for Thrombin-α2macroglobulin Activity
Source: J Clin Med. 2020 Sep 24;9(10):3077. doi: 10.3390/jcm9103077 (PMC7650706; doi:10.3390/jcm9103077)
Supplement: Supplementary file 1 [file jcm-09-03077-s001.zip › Supplemental Figure S3.docx]

**
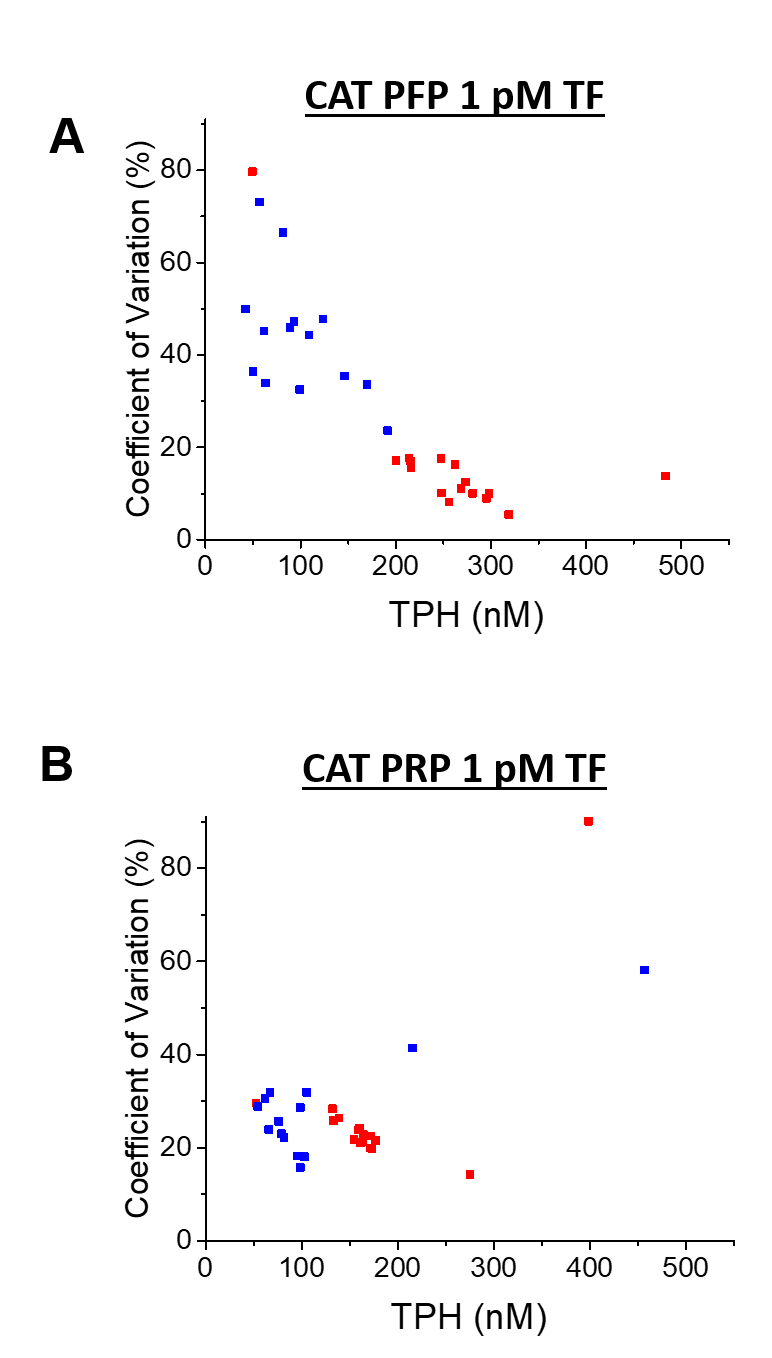
**

**Supplemental Figure S3. Relative adjustment in % of TPH after internal calibration vs. external calibration. Not all runs are shown**. CV’s for TPH values in 1pm TF triggered PFP or PFP with (blue) or without (red) added TM after CAT calibration.
