# Supplementary material for: Comparative Analysis of Thrombin Calibration Algorithms and Correction for Thrombin-α2macroglobulin Activity
Source: J Clin Med. 2020 Sep 24;9(10):3077. doi: 10.3390/jcm9103077 (PMC7650706; doi:10.3390/jcm9103077)
Supplement: Supplementary file 1 [file jcm-09-03077-s001.zip › Supplemental Figure S4.docx]

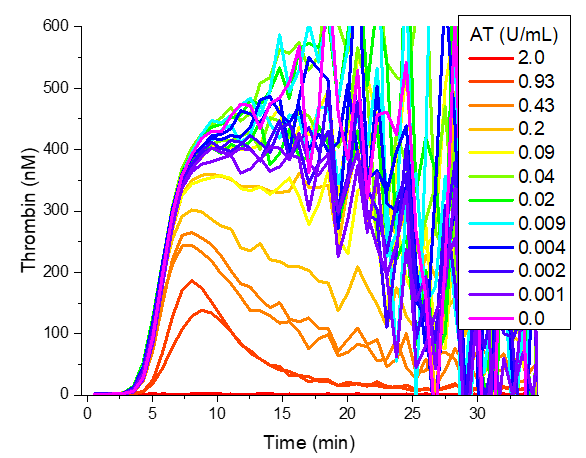


**Supplemental Figure S4. Effect of antithrombin (AT) addition to AT-deficient plasma on TG**. Affinity depleted antithrombin (AT)- deficient platelet free plasma was supplemented with the indicated concentrations of AT concentrate (1U/mL = 100% normal activity) and analyzed with our version of CAT software (allowing for the correction of substrate consumption and the inner filter effect). Increasing concentrations of AT substantially decreases the thrombin peak height.
