# Supplementary material for: Comparative Analysis of Thrombin Calibration Algorithms and Correction for Thrombin-α2macroglobulin Activity
Source: J Clin Med. 2020 Sep 24;9(10):3077. doi: 10.3390/jcm9103077 (PMC7650706; doi:10.3390/jcm9103077)
Supplement: Supplementary file 1 [file jcm-09-03077-s001.zip › Supplemental Table S1 REVISED.docx]

**Supplemental Table S1. Effect of correction algorithms on coefficient of variation for normal plasma samples.** Mean values and coefficients of variation (percent ratio between standard deviation and mean) for TPH are shown.

|  | Calibration and correction algorithms | | Results of TGT analysis | | | |
| --- | --- | --- | --- | --- | --- | --- |
|  | Internal/external calibration | T-α2MG correction | TPH (nM)  PFP 1 pM TF | CV | TPH (nM)  PRP 1 pM TF | CV |
| CAT (CAT software) | Internal | yes | 200 | 17% | 132 | 28% |
| CAT (our software) | Internal | no | 202 | 15% | 147 | 27% |
|  | Internal | yes | 199 | 16% | 139 | 27% |
| Linearly calibrated (our software) | Internal | no | 195 | 17% | 130 | 23% |
|  | External | no | 195 | 17% | 131 | 28% |
