# Supplementary material for: Comparative Analysis of Thrombin Calibration Algorithms and Correction for Thrombin-α2macroglobulin Activity
Source: J Clin Med. 2020 Sep 24;9(10):3077. doi: 10.3390/jcm9103077 (PMC7650706; doi:10.3390/jcm9103077)
Supplement: Supplementary file 1 [file jcm-09-03077-s001.zip › Supplemental Table S2 REVISED.docx]

**Supplemental Table S2: Coefficients of variation for TPHs for all conditions analyzed.** The percent ratio between standard deviations and means for TPH from 6-8 experimental runs of the same condition are shown.

| **Sample** | **Linear calibration** | | **Nonlinear calibration** | | **Nonlinear calibration w/ T-α2MG corrections** | | **CAT** | |
| --- | --- | --- | --- | --- | --- | --- | --- | --- |
|  | **PFP** | **PRP** | **PFP** | **PRP** | **PFP** | **PRP** | **PFP** | **PRP** |
| **NPP** | 16.5% | 22.8% | 15.4% | 27.0% | 15.7% | 27.0% | 17.2% | 28.4% |
| **+100% FII** | 15.1% | 13.1% | 13.9% | 10.6% | 14.3% | 14.8% | 16.3% | 14.3% |
| **+300% FII** | 9.3% | 39.8% | 15.3% | 59.3% | 17.5% | 60.7% | 13.8% | 90.0% |
| **+100% FV** | 15.2% | 17.9% | 14.1% | 21.3% | 14.2% | 22.6% | 15.7% | 25.8% |
| **+300% FV** | 15.5% | 20.6% | 13.5% | 24.5% | 14.3% | 26.3% | 17.0% | 26.4% |
| **+100% FVIII** | 9.0% | 18.3% | 7.0% | 21.8% | 7.2% | 23.3% | 10.0% | 23.8% |
| **+300% FVIII** | 4.5% | 16.6% | 3.0% | 20.3% | 2.9% | 21.4% | 5.5% | 21.6% |
| **+100% FIX** | 9.4% | 18.9% | 8.3% | 23.0% | 8.6% | 23.8% | 8.1% | 22.8% |
| **+300% FIX** | 11.9% | 17.3% | 10.6% | 21.1% | 10.8% | 22.3% | 10.0% | 22.6% |
| **+100% FX** | 16.6% | 16.7% | 15.5% | 18.7% | 15.6% | 20.0% | 17.6% | 21.1% |
| **+300% FX** | 11.7% | 19.6% | 10.1% | 22.5% | 10.2% | 24.1% | 12.5% | 24.2% |
| **+100% FXI** | 11.2% | 18.0% | 10.8% | 21.4% | 11.0% | 21.8% | 10.2% | 21.3% |
| **+300% XI** | 10.2% | 15.9% | 9.3% | 19.2% | 9.4% | 20.6% | 9.0% | 19.9% |
| **+100% FI** | 16.4% | 19.0% | 14.7% | 22.4% | 14.9% | 23.3% | 17.7% | 21.7% |
| **+300% FI** | 11.9% | 17.0% | 11.1% | 21.0% | 11.3% | 21.4% | 11.1% | 19.9% |
| **NPP +TM** | 76.9% | 26.0% | 77.3% | 26.3% | 79.6% | 27.5% | 79.7% | 29.6% |
| **+100% FII +TM** | 42.8% | 38.8% | 43.9% | 39.9% | 44.6% | 40.4% | 44.3% | 41.5% |
| **+300% FII +TM** | 22.0% | 36.0% | 24.0% | 55.7% | 23.6% | 58.0% | 23.6% | 58.1% |
| **+100% FV +TM** | 70.9% | 21.3% | 71.3% | 21.9% | 72.8% | 24.4% | 73.1% | 28.8% |
| **+300% FV +TM** | 64.5% | 27.0% | 65.2% | 26.5% | 66.4% | 28.9% | 66.5% | 30.6% |
| **+100% FVIII +TM** | 45.2% | 18.5% | 47.1% | 19.3% | 47.8% | 20.1% | 470.2% | 22.1% |
| **+300% FVIII +TM** | 31.7% | 13.7% | 34.5% | 15.3% | 34.6% | 15.2% | 33.6% | 15.8% |
| **+100% FIX +TM** | 34.1% | 15.0% | 35.3% | 17.4% | 36.1% | 18.7% | 33.9% | 23.0% |
| **+300% FIX +TM** | 32.8% | 15.3% | 34.8% | 15.6% | 35.1% | 15.8% | 32.5% | 18.2% |
| **+100% FX +TM** | 44.5% | 19.7% | 45.6% | 20.6% | 45.7% | 22.1% | 46.0% | 24.0% |
| **+300% FX +TM** | 33.5% | 22.9% | 34.9% | 24.8% | 35.4% | 24.9% | 35.4% | 25.7% |
| **+100% FXI +TM** | 44.1% | 24.4% | 45.6% | 25.5% | 45.8% | 26.4% | 45.2% | 28.6% |
| **+300% FXI +TM** | 44.9% | 15.2% | 46.9% | 15.7% | 47.0% | 16.9% | 47.7% | 18.1% |
| **+100% FI +TM** | 46.9% | 27.4% | 48.1% | 26.9% | 49.3% | 29.4% | 50.0% | 31.9% |
| **+300% FI +TM** | 38.0% | 29.0% | 39.5% | 31.8% | 39.8% | 32.3% | 36.5% | 31.9% |
